# Clinical and echocardiographic predictors of mortality in acute pulmonary embolism

**DOI:** 10.1186/s12947-016-0087-y

**Published:** 2016-10-28

**Authors:** Talal Dahhan, Irfan Siddiqui, Victor F. Tapson, Eric J. Velazquez, Stephanie Sun, Clemontina A. Davenport, Zainab Samad, Sudarshan Rajagopal

**Affiliations:** 1 Department of Medicine, Division of Pulmonary, Allergy and Critical Care Medicine, Duke University, Durham, NC USA; 2 Department of Medicine, East Carolina University, Greenville, NC USA; 3 Department of Medicine, Division of Pulmonary and Critical Care Medicine, Cedars Sinai Medical Center, Los Angeles, CA USA; 4 Department of Medicine, Division of Cardiology, Duke University, Durham, NC USA; 5 Department of Biostatistics and Bioinformatics, Duke University Medical Center, Durham, NC USA; 6 Center for Pulmonary Vascular Disease, Box 102351, DUMC, Durham, NC 27710 USA

**Keywords:** Echocardiography, Pulmonary embolism, Right ventricular function, Speckle-tracking echocardiography

## Abstract

**Purpose:**

The aim of this study was to evaluate the utility of adding quantitative assessments of cardiac function from echocardiography to clinical factors in predicting the outcome of patients with acute pulmonary embolism (PE).

**Methods:**

Patients with a diagnosis of acute PE, based on a positive ventilation perfusion scan or computed tomography (CT) chest angiogram, were identified using the Duke University Hospital Database. Of these, 69 had echocardiograms within 24–48 h of the diagnosis that were suitable for offline analysis. Clinical features that were analyzed included age, gender, body mass index, vital signs and comorbidities. Echocardiographic parameters that were analyzed included left ventricular (LV) ejection fraction (EF), regional, free wall and global RV speckle-tracking strain, RV fraction area change (RVFAC), Tricuspid Annular Plane Systolic Excursion (TAPSE), pulmonary artery acceleration time (PAAT) and RV myocardial performance (Tei) index. Univariable and multivariable regression statistical analysis models were used.

**Results:**

Out of 69 patients with acute PE, the median age was 55 and 48 % were female. The median body mass index (BMI) was 27 kg/m^2^. Twenty-nine percent of the cohort had a history of cancer, with a significant increase in cancer prevalence in non-survivors (57 % vs 29 %, *p* = 0.02). Clinical parameters including heart rate, respiratory rate, troponin T level, active malignancy, hypertension and COPD were higher among non-survivors when compared to survivors (*p* ≤ 0.05). Using univariable analysis, NYHA class III symptoms, hypoxemia on presentation, tachycardia, tachypnea, elevation in Troponin T, absence of hypertension, active malignancy and chronic obstructive pulmonary disease (COPD) were increased in non-survivors compared to survivors (*p* ≤ 0.05). In multivariable models, RV Tei Index, global and free (lateral) wall RVLS were found to be negatively associated with survival probability after adjusting for age, gender and systolic blood pressure (*p* ≤ 0.05).

**Conclusion:**

The addition of echocardiographic assessment of RV function to clinical parameters improved the prediction of outcomes for patients with acute PE. Larger studies are needed to validate these findings.

## Background

Acute pulmonary Embolism (PE) is a major cause of morbidity and mortality in the United States and Europe, accounting for 100,000 and 300,000 deaths annually, respectively [1, 2]. PE can be classified as massive, submassive or nonmassive based on the hemodynamic status and right ventricular (RV) function of the patient. Massive PE is characterized by systemic hypotension or cardiogenic shock, submassive PE is characterized by RV dysfunction without hypotension, and nonmassive PE has neither systemic hypotension nor RV dysfunction [2]. Massive PE is associated with an in-hospital mortality of 25–50 %, submassive PE with a mortality rate of 3–15 %, while nonmassive PE is associated with mortality of 5 % or less [3]. Risk assessment in patients with submassive PE can be difficult, as the mortality rates for submassive PE can approach that of massive PE [4]. While there is a consensus that systemic thrombolysis, catheter-directed interventions, or surgery are indicated in patients with massive PE, the management of patients with submassive PE remains controversial. Therefore, there remains a challenge in the clinical management of patients who have stable hemodynamics but demonstrate evidence of RV dysfunction, either by electrocardiogram, echocardiogram, computed tomography (CT) scan or cardiac biomarkers [2]. The benefit of thrombolytic or invasive therapies relative to the risk of bleeding is unclear among such patients [5]. An improved approach to risk assessment could therefore allow the identification of those patients presenting with submassive PE who would benefit most from therapy.

To address this, risk assessments based on clinical and imaging parameters have been developed. The Pulmonary Embolism Severity Index (PESI) is an excellent clinical predictor of outcomes in patients with PE [6]. It is based on 11 clinical criteria including age, sex, history of cancer and hemodynamic parameters. Five risk categories are included, ranging from very low risk, with 30-day mortality of less than 2 %, to very high risk, with 10.0–24.5 % mortality [6]. The simplified PESI (sPESI) was subsequently developed, with only six, rather than 11, clinical criteria. In this index, only two risk categories were included, with low risk associated with 1.1 % mortality and high risk associated with an 8.9 % risk of death [7]. Quantitative echocardiographic assessment has been gaining importance in patients with RV dysfunction, including those with congenital heart disease, pulmonary hypertension and pulmonary embolism [8, 9]. A number of studies [10–21] have tested the utility of novel echocardiographic or serum biomarkers for risk assessment in acute PE, but only a few studies have tested whether such parameters provide additional benefit to clinical predictors [22, 23]. We hypothesized that the addition of quantitative echocardiographic markers of RV function would add to clinical parameters to predict outcomes in patients with acute PE.

## Methods

### Study population

We retrospectively identified patients who had a diagnosis of acute PE between January 2010 and April 2014, confirmed by contrasted computed tomography (CT) scan of the chest and/or ventilation-perfusion (VQ) nuclear medicine imaging at Duke University Medical Center (Durham, NC, USA) using the Duke Enterprise Data Unified Content Explorer (DEDUCE) [24]. Subjects were included in the study if they had an echocardiogram performed within 24–48 h of diagnosis of acute PE. Subjects were excluded if their echocardiographic images were suboptimal for RV strain measurement due to poor image quality or poor RV views. Clinical data including demographics, medical history, comorbidities, systemic blood pressure, heart rate, respiratory rate, oxygen saturation on room air and with supplemental oxygen, were collected from the medical record. The Duke University Medical Center Institutional Review Board approved this study.

### Echo-derived parameters of RV function

**Fig. 1:**
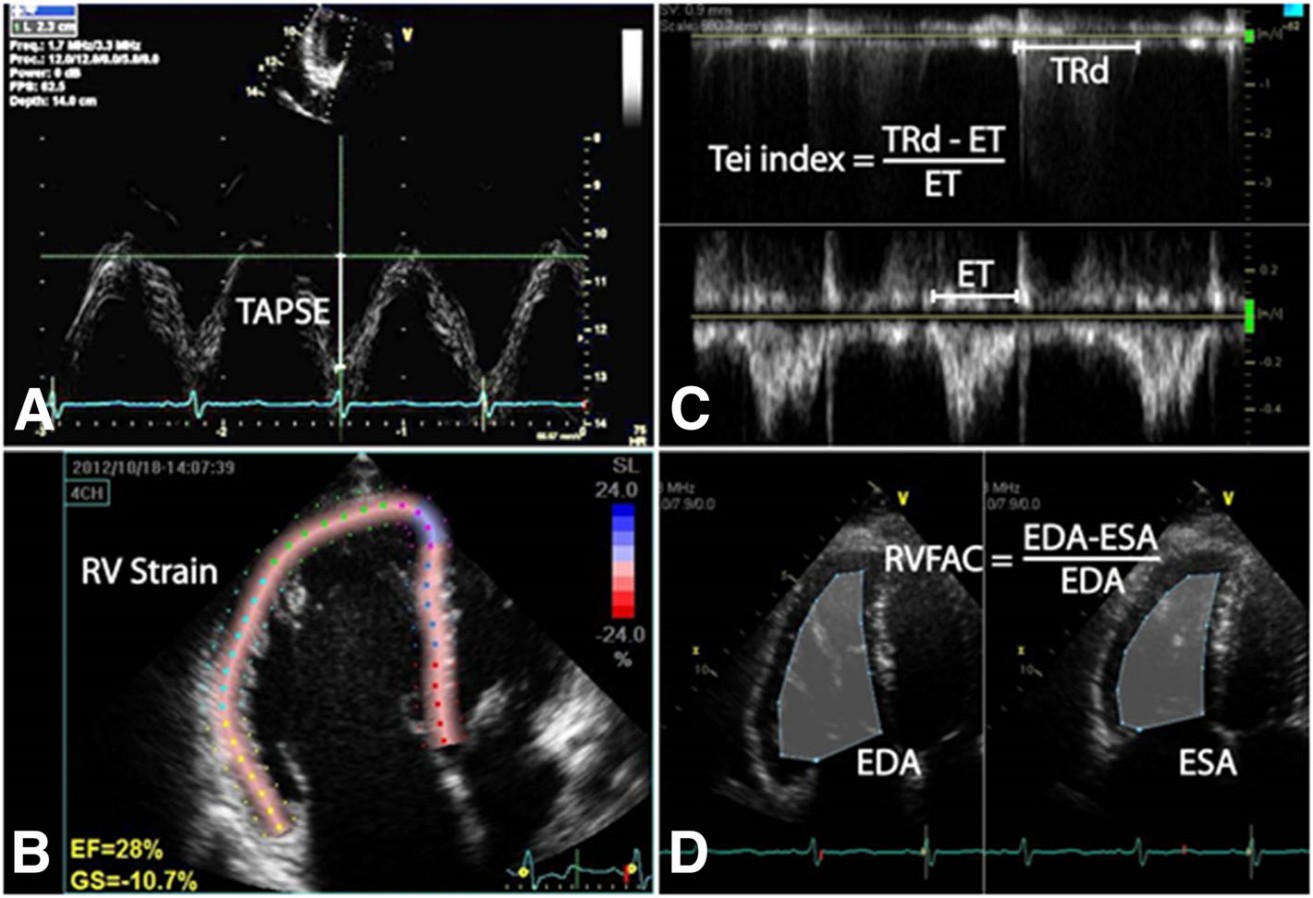
Different 2D echocardiographic methods used in this study to assess RV function. **a** Tricuspid Annular Plane Systolic Excursion (TAPSE) is determined from an M-mode image through the lateral tricuspid annulus by calculating the amount of longitudinal motion of the tricuspid annulus at peak systole. **b** RV longitudinal strain is calculated from speckle-tracking of an RV focused apical 4-chamber view. **c** RV Tei index is calculated as the RV isovolumic time (equal to tricuspid regurgitation duration (TRd) – ejection time (ET)) divided by the ET using the pulsed Doppler method. **d** RV Fractional Area Change (RV FAC) was calculated as the [(RV end-diastolic area – end-systolic area)/end-diastolic area] × 100

All echocardiographic studies were performed on GE Vivid E9 using a 3.5 MHz probe (GE, Vingmed Ultrasound, Hortom, Norway) or Philips IE33 (Philips, Netherlands). Off-line analyses of images were performed in Xcelera (Philips, Andover, MA) and Image-Arena (TomTec Imaging Systems, Unterschleißheim, Germany) by a single experienced reader and analysis was confirmed by a separate experienced reader; inter-reader variability for these studies have been shown to be low [25]. TAPSE was determined from an M-mode through the lateral tricuspid annulus by calculating the amount of longitudinal motion of the annulus at peak systole [26] (Fig. 1a). RV Tei index was calculated as the RV isovolumic time (IVT) divided by the ejection time (ET) using the pulsed Doppler method [8] (Fig. 1c). IVT was calculated as the duration of tricuspid regurgitation from continuous wave Doppler across the tricuspid valve minus the ET from a single representative beat. ET was calculated as the duration of RV outflow on pulsed Doppler across the RVOT from a single representative beat (Fig. 1c). Care was taken to use beats with similar RR intervals to minimize errors in calculation. RV Fraction Area Change (FAC) [27] was calculated as the [(RV end-diastolic area – end-systolic area)/end-diastolic area] × 100 (Fig. 1d). The RV endocardium was traced in systole and diastole from the annulus, along the free wall, to the apex and back along the interventricular septum using the apical four-chamber view. Attempts were made to trace the free wall beneath trabeculations (Fig. 1d).

### RV longitudinal strain

2D strain analysis was performed from the apical 4-chamber view as previously described [28] (Fig. 1b). The reference point for a single cardiac cycle was placed at the beginning of the QRS. Pulmonic valve closure was determined from the pulsed wave Doppler profile of the RV outflow tract. The endocardial border was traced in end systole and the region of interest was adjusted to exclude the pericardium. The quality of the tracking was confirmed visually from 2D images and from the strain traces. Segments with persistently inadequate tracking despite attempts at improving border definition and region of interest were excluded from analysis. The longitudinal strain of the RV free wall (RV_free_) was calculated as the average of the three free wall segments and the longitudinal strain of the RV septum (RV_sept_) was calculated as the average of the three septal segments. Global RVLS was calculated as the average of strains from all segments. All strain and other 2D echo-derived parameter analyses were performed blinded to clinical data.

### Statistical analysis

Demographic and clinical characteristics of participants were presented in the study by survival status and the two groups were compared using Fisher’s exact test for categorical variables and a Kruskal-Wallis rank sum test for continuous variables. Continuous variables were summarized by the median and interquartile range and categorical variables were summarized by counts and percentages (Tables 1 and 2). Table 3 displays the odds ratios and 95 % confidence intervals resulting from univariable logistic regression modeling the probability of survival. These models investigated the association between clinical characteristics, cardiac biomarkers, specific echocardiographic features and PESI predictor score on the probability of survival and no adjustment for multiple testing was done. Multivariable models were fit to investigate the effects of some clinical features with echocardiographic parameters on survival, and the results are shown in Table 4. Statistical analyses were performed using SAS 9.4 (SAS, Cary, NC) and R (R Core Team (2015), Vienna, Austria).

**Table 1:** Baseline characteristics for the cohort of patients with acute PE

Parameter (n)	All patients	Non-Survivors	Survivors	*p*-Value
	69	14	55	
Age at Diagnosis (69)	55 [43–72]	60 [53.5–69.8]	50 [42.5–72]	0.11
Female Gender (69)	33 (48)	6 (42.86)	27 (49.09)	0.77
Body Mass Index (69)	27 [23–28]	26.5 [22.2–27]	27 [23–28]	0.42
Vital Signs:				
Systolic Blood Pressure (66)	118 [107–137]	111 [99.5–144.8]	118 [109–136.5]	0.38
Diastolic Blood Pressure (66)	73 [61.5–81.5]	65 [60.8–71]	75.5 [63.5–83.8]	0.08
Heart Rate (66)	94 [84–111.5]	115 [102.2–125.5]	90 [81.8–105.8]	0.02
Respiratory Rate (66)	20 [18–24]	24 [21.5–28]	20 [18–24]	0.01
Fraction of Inspired Oxygen (69)	0.2 [0.2–0.3]	0.3 [0.2–0.4]	0.2 [0.2–0.3]	0.01
Troponin T level (ng/mL) (23)	0.1 [0–0.3]	0.6 [0.5–0.7]	0.1 [0–0.2]	<0.01
Medical History				
Essential Hypertension (69)	25 (36.23)	9 (64.29)	16 (29.09)	0.03
Type II Diabetes (69)	4 (5.8)	1 (7.14)	3 (5.45)	>0.99
Hypothyroidism (69)	6 (8.7)	1 (7.14)	5 (9.09)	>0.99
Chronic Kidney Disease (69)	3 (4.35)	1 (7.14)	2 (3.64)	0.5
Previous Venous Thromboembolism (69)	9 (13.04)	0 (0)	9 (16.36)	0.19
Connective Tissue Disease (69)	2 (2.9)	1 (7.14)	1 (1.82)	0.37
Active Malignancy (69)	20 (28.99)	8 (57.14)	12 (21.82)	0.02
Orthopedic Fracture or Injury (69)	5 (7.25)	0 (0)	5 (9.09)	0.58
Chronic Obstructive Pulmonary Disease (69)	5 (7.25)	3 (21.43)	2 (3.64)	0.05
Shortness of Breath:				
NYHA class1	2 (2.9)	0 (0)	2 (3.64)	
NYHA class2	5 (7.25)	1 (7.14)	4 (7.27)	
NYHA class3	15 (21.74)	1 (7.14)	14 (25.45)	
NYHA class4	47 (68.12)	12 (85.71)	35 (63.64)	
Pulmonary Embolism Severity Index (PESI) class (69)				<0.01
Class 1	32 (46.38)	1 (7.14)	31 (56.36)	
Class 2	10 (14.49)	2 (14.29)	8 (14.55)	
Class 3	17 (24.64)	5 (35.71)	12 (21.82)	
Class 4	10 (14.49)	6 (42.86)	4 (7.27)	

Shown are median and inter-quartile range in brackets or number of patients with percent of patients in parentheses. *P*-value denotes comparison between non-survivors and survivors

*Abbreviations*: *NYHA* New York Heart Association

**Table 2:** Echocardiographic parameters in survivors and nonsurvivors with acute PE

	Overall	Non-Survivors	Survivors	*p*-Value
Parameter (n)	69	14	55	
TAPSE (52) (cm)	1.9 [1.5–2.3]	2.1 [1.2–2.4]	1.9 [1.6–2.2]	0.82
Global RVLS (69) (%)	−18.1 [−20.8–14.9]	−15.7 [−19.2–12.1]	−18.6 [−21.8–15.6]	0.05
Average Septal RV Wall strain (69) (%)	−17.8 [−22.7–13.9]	−16.8 [−18.6–9.3]	−17.8 [−22.9–14.7]	0.20
Average Free Wall RV Wall strain (69) (%)	−18.2 [−22.8–13.9]	−15 [−18.7–10.3]	−19.2 [−23.2–14.3]	0.04
RV Tei Index (59)	0.5 [0.4–0.5]	0.5 [0.5–0.6]	0.5 [0.4–0.5]	0.01
Pulmonary Artery Acceleration Time (64) (ms × 10)	9.5 [7–12]	9 [7–10]	10 [7–12]	0.58
RV/LV ratio in systole (52)	1 [0.8–1.4]	1.1 [1–1.4]	1 [0.8–1.5]	0.74
RV/LV ratio in diastole (52)	0.7 [0.6–0.9]	0.8 [0.7–1]	0.7 [0.6–0.9]	0.35
RV base (42) (cm)	3.8 [3.3–4.3]	4.2 [3.5–4.3]	3.7 [3.3–4.3]	0.61
RV middle (39) (cm)	3.3 [2.9–3.6]	3.5 [3–4.5]	3.3 [2.9–3.6]	0.31
RV length (39) (cm)	7.5 [7–7.9]	7.5 [7.2–7.8]	7.5 [6.9–7.9]	0.61
RV diastolic diameter (52) (cm)	2.7 [2.1–3.1]	2.7 [2.3–3.1]	2.7 [2.1–3.2]	0.41
LV diastolic diameter (52) (cm)	3.5 [2.9–4.1]	3.2 [2.9–3.8]	3.6 [3–4.2]	0.63
RV systolic diameter (52) (cm)	2.4 [2.1–2.9]	2.6 [2.2–2.9]	2.4 [2–2.9]	0.57
LV systolic diameter (34) (cm)	2.3 [1.9–2.7]	2.2 [1.9–3]	2.4 [2–2.6]	0.65
RV fractional area change (67) (%)	34.4 [27.6–43.1]	27.9 [19.6–35.8]	38.6 [28.9–43.8]	0.02
RV area in diastole (67) (cm^2^)	24.3 [19.9–31]	25 [22–44]	24.1 [19.3–29.8]	0.22
RV area in Systole (67) (cm^2^)	16.8 [11.4–19.6]	16.8 [14.6–26.3]	16.9 [11.1–19.4]	0.20
RV basal free wall strain (69) (%)	−21.4 [−27.4–13.5]	−14.8 [−23.9–10.9]	−22.4 [−28.2–16.1]	0.06
RV middle free wall strain (69) (%)	−16.3 [−22.6–9.5]	−12.4 [−14.5–9.2]	−17.9 [−23.5–9.8]	0.10
RV apical free wall strain (69) (%)	−13.9 [−21.4–8.3]	−11.4 [−15.2–7.7]	−14.9 [−21.4–9.2]	0.33
RV basal septal strain (69) (%)	−19 [−24.5–13.9]	−17.1 [−20.1–13]	−19.6 [−24.8–13.9]	0.23
RV middle septal strain (69) (%)	−18.4 [−23.3–13.7]	−15.6 [−22.8–8.5]	−19 [−22.8–14.2]	0.32
RV apical septal strain (69) (%)	−16 [−19.7–9.5]	−12.1 [−17.7–7]	−16.4 [−19.9–9.8]	0.15
RV subjective size				0.28
Normal RV size	39 (56.52)	7 (50)	32 (58.18)	
Mild RV dilation	11 (15.94)	3 (21.43)	8 (14.55)	
Moderate RV dilation	12 (17.39)	1 (7.14)	11 (20)	
Severe RV dilation	7 (10.14)	3 (21.43)	4 (7.27)	
RV subjective function				0.60
Normal RV function	38 (55.07)	6 (42.86)	32 (58.18)	
Mildly reduced RV function	10 (14.49)	2 (14.29)	8 (14.55)	
Moderately reduced RV function	15 (21.74)	4 (28.57)	11 (20)	
Severely reduced RV function	6 (8.7)	2 (14.29)	4 (7.27)	
LV ejection fraction (69)				0.07
LV ejection fraction < 40 %	6 (8.7)	3 (21.43)	3 (5.45)	
LV ejection fraction 40–50 %	6 (8.7)	2 (14.29)	4 (7.27)	
LV ejection fraction >50 %	57 (82.61)	9 (64.29)	48 (87.27)	

Shown are median and inter-quartile range in brackets or number of patients with percent of patients in parentheses. *P*-value denotes comparison between non-survivors and survivors

*Abbreviations*: *RV* right ventricle, *LV* left ventricle, *TAPSE* tricuspid annular plane systolic excursion, *RVLS* RV longitudinal strain

**Table 3:** Univariable analysis of clinical and echocardiographic parameters in predicting outcome in acute PE

	OR	2.5 %	97.5 %	Estimate	SE	Z	*p*-value
Age at Diagnosis (69) (years)	0.98	0.94	1.01	−0.02	0.02	−1.44	0.15
Female Gender (69)	1.29	0.39	4.20	0.25	0.60	0.42	0.68
Body Mass Index (69) (kg/m^2^)	1.06	0.93	1.20	0.06	0.06	0.87	0.39
Systolic blood pressure (66) (mmHg)	1.01	0.99	1.04	0.01	0.01	0.91	0.36
Diastolic blood pressure (66) (mmHg)	1.04	1.00	1.10	0.04	0.02	1.80	0.07
Malignancy (69)	0.21	0.06	0.72	−1.56	0.63	−2.48	0.01
Troponin T (23) (ng/mL)	0.00	0.00	0.52	−13.18	6.39	−2.06	0.04
CK-MB (50) (ng/mL)	0.99	0.93	1.04	−0.01	0.03	−0.52	0.60
Mild subjective RV dilation (69)	0.58	0.12	2.77	−0.54	0.80	−0.68	0.50
Moderate subjective RV dilation (69)	2.41	0.27	21.81	0.88	1.12	0.78	0.43
Severe subjective RV dilation (69)	0.29	0.05	1.61	−1.23	0.87	−1.42	0.16
Mild subjective RV dysfunction (69)	0.75	0.13	4.44	−0.29	0.91	−0.32	0.75
Moderate subjective RV dysfunction (69)	0.52	0.12	2.17	−0.66	0.73	−0.90	0.37
Severe subjective RV dysfunction (69)	0.37	0.06	2.53	−0.98	0.97	−1.01	0.31
LV ejection fraction 40–50 % (69)	2.00	0.19	20.61	0.69	1.19	0.58	0.56
LV ejection fraction > 50 %(69)	5.33	0.93	30.74	1.67	0.89	1.87	0.06
Global RVLS (69) (%)	0.88	0.78	0.99	−0.13	0.06	−2.13	0.03
Free wall RV strain (69) (%)	0.90	0.82	1.00	−0.10	0.05	−2.00	0.05
TAPSE (52) (cm)	1.47	0.43	4.96	0.38	0.62	0.62	0.54
RV/LV ratio in systolic (52)	1.10	0.23	5.22	0.10	0.79	0.12	0.90
RV/LV ratio in diastolic (52)	0.39	0.02	6.50	−0.95	1.44	−0.66	0.51
RV Tei index (59)	0.00	0.00	0.34	−7.00	3.02	−2.32	0.02
RVFAC (67)	1.05	1.00	1.12	0.05	0.03	1.81	0.07
PAAT (64) (ms × 10)	1.07	0.89	1.30	0.07	0.10	0.72	0.47
PESI class 3 (69)	0.13	0.01	1.61	−2.05	1.29	−1.59	0.11
PESI class 4 (69)	0.08	0.01	0.73	−2.56	1.15	−2.23	0.03
PESI class 5 (69)	0.02	0.00	0.23	−3.84	1.20	−3.19	<0.01

Odds ratio (survival over non-survival), 95 % confidence interval, estimate, standard error, and *p*-value of the univariable logistic regression models are shown

*Abbreviations*: *CK-MB*, creatine kinase-myocardial band, *RV* right ventricle, *LV* left ventricle, *RVLS* RV longitudinal strain, *TAPSE* tricuspid annular plane systolic excursion, *RVFAC* RV fractional area change, *PAAT* pulmonary artery acceleration time, *PESI* pulmonary embolism severity index

**Table 4:** Multivariable analysis of clinical and echocardiographic parameters in predicting outcome in acute PE

	OR	2.5 %	97.5 %	Estimate	SE	Z	*p*-value
Global RVLS model							
Age	0.99	0.95	1.02	−0.01	0.02	−0.74	0.46
Female Gender	1.20	0.32	4.58	0.18	0.68	0.27	0.79
Systolic Blood Pressure	1.01	0.99	1.04	0.01	0.01	0.97	0.33
Global RVLS	0.87	0.77	1.00	−0.14	0.07	−2.01	0.04
Free wall RVLS model							
Age	0.99	0.95	1.03	−0.01	0.02	−0.68	0.50
Female Gender	1.21	0.32	4.61	0.19	0.68	0.28	0.78
Systolic Blood Pressure	1.01	0.98	1.04	0.01	0.01	0.67	0.50
Free wall RVLS	0.89	0.80	1.00	−0.11	0.06	−1.95	0.05
RV Tei Index model							
Age at Diagnosis	0.99	0.95	1.03	−0.01	0.02	−0.53	0.60
Female Gender	0.78	0.17	3.47	−0.26	0.76	−0.33	0.74
Systolic Blood Pressure	1.02	0.99	1.05	0.02	0.02	1.19	0.23
RV Tei Index	0.00	0.00	0.36	−7.82	3.46	−2.26	0.02

Odds ratio (survival over non-survival), 95 % confidence interval, estimate, standard error, and *p*-value of the multivariable logistic regression models are shown

## Results

During the study period, 135 patients were admitted with a clinical diagnosis of acute PE. Among these, 95 patients diagnosed with an acute PE had a transthoracic echocardiogram within the initial 24–48 h of admission. 26 patients did not have suitable images for offline analysis, resulting in a cohort of 69 analyzed subjects. Six of the subjects underwent thrombolysis. At 30 days, of these 69 subjects, 14 had died and 55 survived. (Table 1).

### Baseline characteristics and presentation

The baseline characteristics of all 69 patients are listed in Table 1. The median age was 55 years old (range 16–95) and 48 % (*n* = 38) of patients were females. The median body mass index (BMI) was 27 kg/m^2^ (range 20–68). With respect to comorbidities, 29 % (*n* = 20) of the cohort had a history of cancer, with a significantly higher prevalence in non-survivors compared to survivors (57 % vs. 29 %, *p* = 0.02). The only other significant difference in comorbidities between non-survivors and survivors was in the prevalence of hypertension (non-survivors, 64 %, vs. survivors, 25 %, *p* = 0.03). 13 % of patients (*n* = 9) had a history of prior venous thromboembolism. On presentation, 90 % (*n* = 62) had NYHA class III symptoms (Table 1). Hypoxemia on presentation, tachycardia, tachypnea, elevation in Troponin T, absence of hypertension, active malignancy and chronic obstructive pulmonary disease (COPD) were statistically significantly different (*p* ≤ 0.05) between non-survivors and survivors (Table 1).

### Echocardiographic assessment of RV function in acute PE

**Fig. 2:**
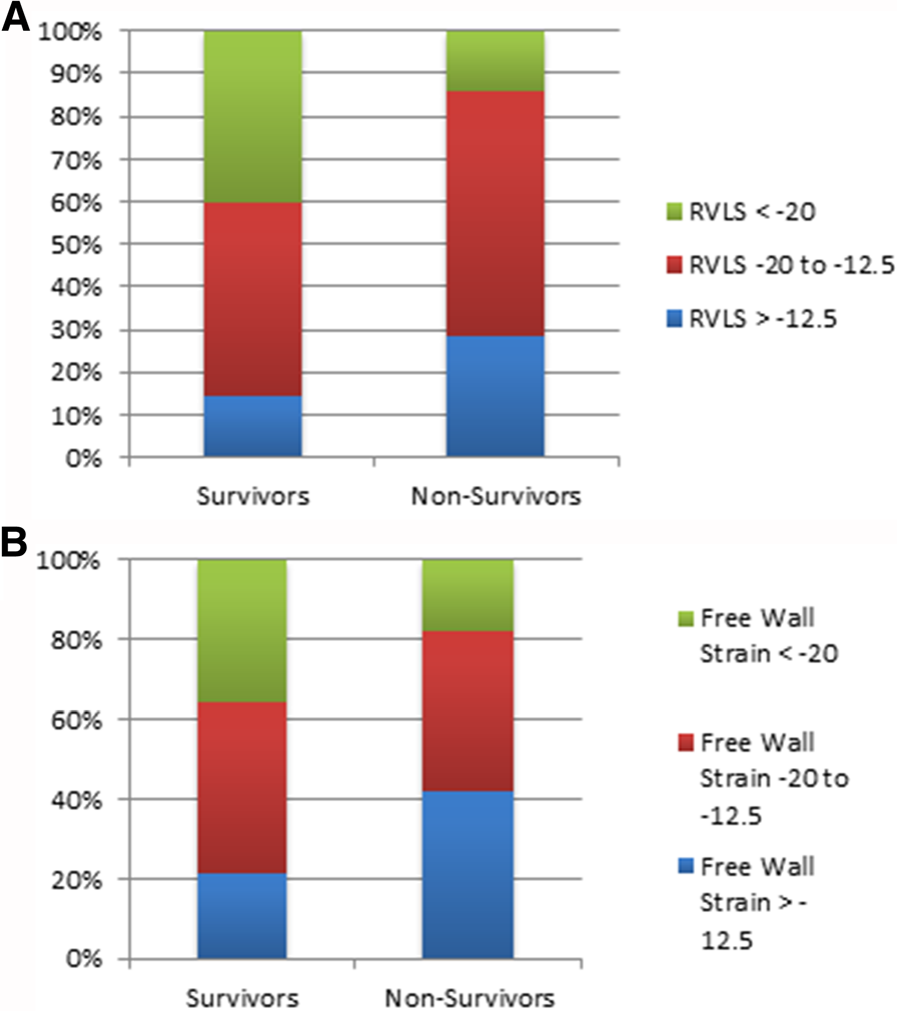
**a** Global Right Ventricular Strain (RVLS) and **b** free wall RVLS categorized as mild, moderate and severe among survivors and non-survivors in a cohort of patients with acute pulmonary embolism

A number of echocardiographic parameters were assessed in our cohort (Table 2). These included parameters that are thought to quantify RV systolic function (TAPSE, global, regional and free wall RV longitudinal strain (RVLS), RV myocardial performance (Tei) Index, RV fraction area change (RVFAC), and subjective echocardiographic evaluation of RV function) and RV size (RV/LV ratio in systole and diastole, diameters of RV and LV in systole and diastole). RVFAC, Tei Index, global and free wall RVLS were significantly different between survivors and nonsurvivors (*p* ≤ 0.05). For example, a significant proportion of non-survivors had global and free wall RVLS of more than −12.5 (Fig. 2), a value which has been demonstrated to be associated with worse outcomes in pulmonary hypertension [29]. TAPSE, subjective RV dilation and subjective RV dysfunction were not statistically different between survivors and non-survivors (*p* > 0.05).

### Univariable and multivariable predictors of outcome in acute PE

On univariable analysis, a number of clinical and echocardiographic parameters were statistically significantly (*p* ≤ 0.05) associated with 30 day mortality. These included active malignancy, serum troponin, global and free wall RVLS, RV Tei Index, and patients with PESI classes four and five. For multivariable regression analysis, we attempted to include PESI as a clinical predictor with selected echo parameters, but could not because PESI score (whether categorical or continuous) demonstrated non-trivial collinearity with global and free wall RVLS and RV Tei index, resulting in unstable standard errors of estimates. Similarly, both heart rate and systolic blood pressure displayed significant collinearity, so only systolic blood pressure was included as it was a predictor in the univariable model. As the multivariable models could not include those clinical and echocardiographic predictors together, we instead used a multivariable model that included parameters used to calculate PESI [6], namely age, gender and systolic blood pressure. With this multivariable regression model, global and free wall RVLS and RV Tei index all predicted outcome with statistical significance (*p* ≤ 0.05).

## Discussion

This study demonstrates that the addition of selected echocardiographic estimates of RV function to clinical parameters in patients with acute PE improved prediction of 30-day mortality in a cohort of patients with acute PE. In our cohort, global, free wall RVLS and RV Tei index analyses were independently associated with mortality on univariable and multivariable analysis. However, other assessments of RV function, including TAPSE, RVFAC, and subjective evaluations of RV size and function were not associated with mortality on univariable analysis. At this time, there are no clear guidelines as to which parameters should be used to assess RV function [8], and significant inter-rater variability exists in subjective evaluation of the RV [9]. The objective echocardiographic assessment of RV function with qualitative parameters, such as RVLS, may reduce inter-rater variability [25] and have utility in identifying submassive PE patients who may benefit most from consideration of aggressive therapies.

The European Society of Cardiology [2] and the American College of Chest Physicians guidelines [30] emphasize the importance of the assessment of RV function and cardiac biomarkers in risk assessment of acute PE, as they may allow the identification of high-risk patients before they clinically deteriorate. An alternative strategy has been the use of clinical risk prediction algorithms, such as PESI and sPESI [6, 7]. In our analysis, we found significant collinearity between PESI and RVLS and Tei index, suggesting that these echocardiographic and clinical parameters are all associated with high risk features. While global and free wall RVLS require special software and analysis to obtain, RV Tei index is relatively straightforward to acquire and could be used broadly. Vitarelli et al. found an association of a number of parameters of RV function with 6 month adverse outcomes in acute PE patients on univariate analysis [27]. Moreover, they found that mid-free wall RVLS, RVSP and 3D RV ejection fraction were associated with adverse outcome on multivariate analysis. While we observed large absolute numerical differences in basal free wall strain between survivors and nonsurvivors, they did not reach statistical significance due to large variance. It is likely that they would have been significant in a larger study. Overall, the results here extend the findings of Vitarelli et al., as we found that both global and free wall RVLS were associated with outcome on multivariable analysis after accounting for age, gender and systolic blood pressure.

While subjective RV dysfunction has been associated with worse outcomes in PE [31], a number of studies suggest that quantifiers of RV function may better identify high-risk patients, although most of these studies did not test the utility of such parameters in combination with clinical characteristics [11, 13–21]. For example, RV dysfunction, as assessed by tricuspid annular plane systolic excursion (TAPSE) and RV myocardial performance (Tei) index, has been characterized in patients with PE [32, 33]. Another recent study identified the ratio of RV to LV end-diastolic diameter, RV systolic pressure, tricuspid annular plane systolic excursion, and inferior vena cava collapsibility to be independently associated with mortality in patients presenting with acute PE [11]. Abnormal RV global and free wall speckle-tracking strains have been associated with adverse events in patients with PE [27]. Ozsu and coworkers demonstrated a correlation of Tei index with treatment response in acute PE [33]. Park and coworkers demonstrated that TAPSE correlated with other parameters of RV function and BNP in acute PE [32]. RV ejection fraction and regional mid wall strain has been prospectively assessed in patients with PE, with a potential to assess a therapeutic response [27]. Thus, there are a number of parameters of RV function that have been shown to correlate with outcomes in acute PE.

## Conclusions

 Although our cohort of patients is small, we found an association of free-wall, global RV strain and RV Tei index with mortality. Notably, we did not find such a relationship between other measures, including TAPSE, RV size and RV/LV ratio. The associations we found were still significant after the inclusion of clinical risk factors [6, 7]. These findings suggest that the addition of echocardiographic parameters to clinical parameters may improve risk prediction in acute PE.

## Limitations

Our study is a retrospective, single center study with a small cohort of patients, which may limit the generalizability of these findings. Patient care was variable, resulting in significant differences in studies that were performed, such as troponin T, which was only performed in 23 of the 69 subjects included in the final analysis. As there was no set protocol for RV imaging in this retrospective study, there was a relatively low suitability (72.6 %) of images for offline analysis and poor tracking observed in the basal and mid-free wall segments. In our analysis, we found significant collinearity between PESI and RVLS and Tei index, preventing us from combining our echo predictors with PESI score. Therefore, it is possible that echo predictors do not add significant information to the PESI score. Previous studies from our group have demonstrated small inter- and intra-observer variability in assessment of RV strain [34], so such analyses were not included in this study. Future prospective, multi-center studies that address objective RV function assessment in relation to outcomes in patients with PE are needed to validate these findings.

## Abbreviations

2D: Two dimensional
3D: Three dimensional
BNP: Brain natriuretic peptide
COPD: Chronic obstructive pulmonary disease
CT: Computed tomography
DEDUCE: Duke Enterprise Data Unified Content Explorer
EF: Ejection fraction
ET: Ejection time
GE: General electric
IVT: Iso volumic time
LV: Left ventricle
PAAT: Pulmonary artery acceleration time
PE: Pulmonary embolism
PESI: Pulmonary embolism severity index
RV: Right ventricle
RVFAC: Right ventricular fraction area change
RVLS: Right ventricular longitudinal strain
RVOT: Right ventricular outflow tract
SAS: Statistical analysis system software
sPESI: simplified pulmonary embolism severity index
TAPSE: Tricuspid annular plane systolic excursion
VQ: Ventilation perfusion
